# Clinical features of uterine sarcomas presenting mainly with uterine masses: a retrospective study

**DOI:** 10.1186/s12905-023-02517-7

**Published:** 2023-07-26

**Authors:** Menghan Zhu, Shouzhen Chen

**Affiliations:** Department of Gynecology, Obstetrics and Gynecology Hospital, Fudan University, Shenyang Road 128, Shanghai, 200090 China

**Keywords:** Uterine sarcoma, Uterine mass, Ultrasound

## Abstract

**Background:**

Uterine sarcomas are uncommon mesenchymal tumors of the uterus. The clinical problem is that the features of uterine sarcomas can sometimes mimic uterine fibroids. This study aims to investigate the clinical characteristics of patients with uterine sarcomas who were preoperative presenting mainly with uterine masses.

**Methods:**

A retrospective analysis of patients who underwent gynecological surgery for uterine sarcomas at the Obstetrics & Gynecology Hospital of Fudan University, between January 2016 and December 2021.

**Results:**

Over the 5-year period, 277 patients were final diagnosed of uterine sarcomas. A total of 162 patients were preoperatively diagnosed as uterine fibroids for surgical treatment, the majority of whom were diagnosed of uterine leiomyosarcoma (uLMS) (49/162) and low-grade endometrial stromal sarcoma (LG-ESS) (100/162). Ninety people underwent total hysterectomy and bilateral salpingo-oophorectomy (TH + BSO), while 72 underwent myomectomy followed by supplemental TH + BSO. The group with direct hysterectomy had a higher average age than the group with prior myomectomy (47.20 ± 8.94 vs. 40.86 ± 5.88, p < 0.001). Among patients preoperatively diagnosed as uterine fibroids, patients with uLMS had a higher proportion of previous myomectomy (26.53% vs. 5.00%, p < 0.001), a larger uterine mass diameter on ultrasound (8.38 ± 3.39 cm vs. 6.41 ± 1.92 cm, p < 0.001), and richer hypervascularity (34.69% vs. 18%, p = 0.024) compared with LG-ESS.

**Conclusions:**

Analysis of our data showed that a large proportion of uterine sarcomas, especially uLMS and LG-ESS, present mainly with uterine masses. Ultrasound features including a large uterine mass diameter and rich hypervascularity, and with a history of myomectomy may alert clinicians in suspicion of uLMS when compared with LG-ESS.

## Background

Uterine sarcomas are uncommon mesenchymal tumors of the uterus, account for 8% of all uterine malignancies [1]. Uterine sarcomas can be divided into four categories: uterine leiomyosarcoma (uLMS), low-grade endometrial stromal sarcoma (LG-ESS), high-grade endometrial stromal sarcoma (HG-ESS), adenosarcomas and high-grade undifferentiated sarcoma (HGUS). There are other rare sarcomas, including perivascular epithelioid cell tumor (PEComa) and rhabdomyosarcoma (RMS). Uterine sarcomas are a heterogeneous group of tumors that are characterized by aggressive clinical behavior. The survival rate variated based on histopathological type. Leiomyosarcomas have an unfavorable prognosis even in apparently early stage. Low-grade endometrial stromal sarcomas have a favorable prognosis associated with long-term survival. High-grade endometrial stromal sarcomas and undifferentiated sarcomas behave more aggressively. Adenosarcomas have a favorable prognosis without sarcomatous overgrowth, and those with sarcomatous overgrowth are associated with aggressive clinical behavior [2]. Nevertheless, uterine sarcomas are often with a high local recurrence rate and metastatic risk, especially among patients in the advanced stages [3].

For uterine sarcomas, a total hysterectomy should be performed [4]. However, the clinical problem is that the features of uterine sarcomas can sometimes mimic uterine fibroids. In case of absence of preoperative diagnosis or suspicion of uterine sarcomas, patients who have a strong desire for uterus preservation often undergo laparoscopic tumorectomy and morcellation of the sarcoma. Hence the risk of tumor dissemination would be elevated by dispersal in tumorectomy and morcellation, which strongly impacts prognosis [5, 6]. The incidence of uLMS and LG-ESS is relatively high in sarcomas, and the prognosis of uLMS is worse than that of LG-ESS. Therefore, it is more important to identify uLMS from uterine masses in order to avoid morcellation.

Recent application of molecular techniques has identified numerous lesions with distinctive genetic abnormalities and clinicopathological characteristics [7], for instance, JAZF1::SUZ12 fusion in LG-ESS, YWHAE::NUTM2 fusion and ZC3H7B::BCOR fusion in HG-ESS, and DICER1 mutations in embryonal RMS. However, unlike cancers of the uterine corpus and cervix, uterine sarcomas are difficult to biopsy, highlighting the importance of improving the accuracy of preoperative diagnosis from clinical features.

This study compared the clinical and ultrasound features of patients with uterine sarcoma presenting mainly with uterine masses. The aim of the present study was to provide evidence for preoperative assessment of uterine masses and hence more effective preoperative identification of different types of uterine sarcomas.

## Methods

Retrospective data collection was done of patients who had undergone gynecological surgery for uterine sarcoma at the Obstetrics & Gynecology Hospital of Fudan University, a single tertiary institute in Shanghai, China, between January 01, 2016 and December 31, 2021. We identified uterine sarcomas by tumor ICD-O code from the electronic medical record system (8890/3 for uLMS, 8931/3 for LG-ESS, 8930/3 for HG-ESS, 8933/3 for adenosarcoma, and 8805/3 for HGUS). Patients with previous history of uterine malignant tumors or concurrent other malignant tumors or multiple primary tumors were excluded. Gynecologic ultrasound (transvaginal, transrectal and/or transabdominal) was performed preoperatively by the same team of expert radiologists who dedicated full time to ultrasound diagnosis of gynecological pathology. In suspected cases, magnetic resonance imaging (MRI) was performed preoperatively by a team of expert radiologists. The histological diagnosis of sarcomas was confirmed by two senior gynecologic pathologists following postsurgical pathology review. The study was approved by the ethics committee of the Obstetrics and Gynecology Hospital of Fudan University. Clinical characteristics were collected, including age, menstrual period, history, preoperative imaging, and surgical approach.

Analysis of the data was performed with IBM^©^ SPSS^®^ 20 software for Windows (SPSS Inc., Chicago, IL, USA). The t-test was used to assess differences in continuous variables between the groups. To quantify the correlation between categorical variables and continuous clinical variables, Pearson χ^2^ test was used. All statistical tests were two-sided. The results were considered statistically significant at p < 0.05.

## Results

Over the 5-year study period, 277 patients were final diagnosed of uterine sarcoma. A total of 162 patients with a preoperatively diagnosis of uterine masses, which were presumed as uterine fibroids, were identified (Table 1). The mean age at diagnosis was 44 years. The average tumor diameter identified by ultrasound was 7.26 ± 2.79 cm. Eighty-two patient had preoperative serum tumor markers assessment. Serum CA125 was elevated in 14 patients (14/82). Thirty patients underwent MRI, 10 of whom were preoperatively diagnosed with suspicious uterine sarcoma. Ninety people underwent total hysterectomy and bilateral salpingo-oophorectomy (TH + BSO), while 72 underwent myomectomy followed by supplemental TH + BSO. The group with direct hysterectomy had a higher average age than the group with prior myomectomy (47.20 ± 8.94 vs. 40.86 ± 5.88, p < 0.001). The majority of these 162 patients were diagnosed of uLMS (49/162) and LG-ESS (100/162). Among these 162 patients, 148 follow-up records were available. Median follow-up length was 24 months (range, 2–124 months). Twenty-six out of 148 experienced recurrence within 17 months in average. The pathological types of these 26 people were: 12 uLMS, 6 LG-ESS, 3 HG-ESS, 1 adenosarcomas, and 4 HGUS. Adjuvant therapy (chemotherapy, radiotherapy, endocrine therapy) was indicated according to prognostic factors such as tumor grade, maximum dimension of the uterine sarcoma, and tumor extension outside the uterus.

**Table 1:** Characteristics of women with uterine sarcoma presenting mainly with uterine masses

Characteristic	n = 162
Age, years	44 ± 8
Menopause	
Yes	137(84.57%)
No	25(15.43%)
History of prior myomectomy	
Yes	22(13.58%)
No	140(86.42%)
Family history of malignancy	
Yes	27(16.67%)
No	135(83.33%)
Menstrual changes or irregular vaginal bleeding	
Yes	44(27.16%)
No	118(72.84%)
Ultrasound tumor diameter(cm)	7.26 ± 2.79
Preoperative diagnosis	10(6.17%)
Laparotomic surgery	38(23.46%)
Myomectomy prior to TH + BSO	72(44.44%)
Histological diagnosis	
uLMS	49(30.25%)
LG-ESS	100(61.73%)
HG-ESS	6(3.70%)
Adenosarcomas	2(1.23%)
HGUS	5(3.09%)
Adjuvant chemotherapy	45(27.78%)
Adjuvant radiotherapy	10(6.17%)
Endocrine therapy	40(24.69%)

Among all patients with uLMS, the proportion (49/56) was the highest regarding uterine masses as the first manifestation, followed by the proportion among LG-ESS (100/156). Forty-five out of 49 uLMS were diagnose with stage IA/IB disease, the remain 4 uLMS patients were diagnosed with stage IIB. The recurrence of uLMS of 11 (11/49) patients with stage IA/IB diseases occurred within 13 months in average. Ninety-three out of 100 LG-ESS were diagnosed with stage IA/IB disease, the remain 7 LG-ESS patients were diagnosed with stage IIB. The recurrence of LG-ESS of 4 (4/100) patient with stage IA/IB diseases occurred within 34 months in average. During the limited follow-up period of this study, the recurrence time of uLMS was earlier than that of LG-ESS, and the recurrence rate was higher than that of LG-ESS.

Among patients preoperatively diagnosed as uterine fibroids, patients with uLMS had a higher proportion of previous myomectomy (26.53% vs. 5.00%, p < 0.001), a larger uterine mass diameter on ultrasound (8.38 ± 3.39 cm vs. 6.41 ± 1.92 cm, p < 0.001), and richer hypervascularity (34.69% vs. 18%, p = 0.024) compared with LG-ESS (Table 2).

**Table 2:** Characteristics of women diagnosed of uLMS or LG-ESS with uterine masses presenting mainly with uterine masses

Characteristics	uLMS(n = 49)		LG-ESS(n = 100)	p
Age, years	44 ± 8		44 ± 8	0.834^a^
Menopause				
Yes	8		13	0.584^b^
No	41		87	
History of myomectomy				
Yes	13		5	< 0.001 ^b^
No	36		95	
Menstrual changes or irregular vaginal bleeding				
Yes	16		22	0.161 ^b^
No	33		78	
Ultrasound tumor diameter(cm)	8.38 ± 3.39		6.41 ± 1.92	< 0.001 ^a^
Heterogeneous echotexture of uterine mass on ultrasound				
Yes	23		46	0.914 ^b^
No	26		54	
Hypervascularity of uterine mass on ultrasound				
Yes	17		18	0.024 ^b^
No	32		82	
Mass affects the uterine cavity on ultrasound				
Yes	7		14	0.962 ^b^
No	42		86	

^a^t test

^b^Pearson χ2 test

## Discussion

As uterine neoplasm, uterine sarcoma tends to be more difficult the identified when compared to endometrial carcinoma. Endometrial carcinoma is more likely to be detected due to abnormal vaginal bleeding. The clinical presentation of uterine sarcomas is nonspecific, including vaginal bleeding, pelvic mass, and abdominal or pelvic pain [1]. Uterine sarcoma is challenging to accurately diagnosed preoperatively and can mimic the appearance of benign uterine leiomyoma. In this study, uLMS was at the highest proportion presenting mainly with uterine masses among uterine sarcomas.

Of the 162 in this study, most of these cases were radiographically suggestive of uterine leiomyoma degeneration. However, it’s notable that tumor diameter on ultrasound reached 7.26 ± 2.79 cm in average. In particular, uLMS reaches a diameter of 8.38 ± 3.39 cm. This suggests that clinicians would be alert to a higher potential for malignancy in large lesions. In patients presenting mainly with uterine masses, we found that uLMS had richer hypervascularity than LG-ESS. Meanwhile, uLMS showed a low proportion of uterine cavity affection on ultrasound that LG-ESS, which may explain the absence of specific symptoms of vaginal bleeding in uLMS. Our findings consist with suspicious imaging features in of uterine sarcomas including a large-size, heterogeneous echotexture, central cystic change or necrosis, and hypervascularity [8]. What remains to be noted is that several imaging modalities have been evaluated for detection of uterine sarcomas, including Doppler ultrasound, computed tomography, and MRI, but none has shown superiority [9, 10].

In this study, 13 out of the 22 patients with a history of myomectomy were diagnosed of uLMS, suggesting that clinicians would be alert to a higher potential for malignancy in patients with large uterine masses with a history of myomectomy. Since variants of benign uterine fibroids may contain changes include hypercellularity, necrosis, nuclear atypia, mitotic figures, and intravascular growth, the distinction between fibroids and uLMS may at times be problematic [11]. The combination of evaluation of conventional morphologic criteria with immunohistochemistry is of great value in the assessment of uterine smooth muscle tumors histology [12]. Since previous myomectomy specimens cannot be all re-evaluated, we would only make one hypothesis that uLMS tumor cells may have been present in previous uterine fibroids, and residual cells may progress to uLMS.

Surgery is considered the mainstay of treatment for uterine sarcomas, and it should be done in order to remove the uterus intact [6, 13]. In case of absence of preoperative diagnosis or suspicion of uterine sarcomas, patients with uterine masses are often treated as uterine fibroids. Surgical treatment of uterine fibroids could be performed by traditional laparotomy, vaginally or with minimally invasive surgery. Minimally invasive treatment of uterine fibroids requires morcellation to remove fibroids through a small excision. Morcellation can be manual or instrumental (power morcellation), both of which can affect patient prognosis [14]. The US Food and Drug Administration (FDA) warned about the use of morcellation in 2014 because of the risk of undiagnosed uLMS in women undergoing morcellation during laparoscopic hysterectomies and myomectomies [15]. The technique of power morcellation in a bag was recently suggested to minimize the risk of inadvertent tissue spread [16]. The 2017 Agency for Healthcare Research and Quality meta-analysis stated that in the case of uterine sarcoma, the expected five-year survival was 30% for women undergoing power morcellation (95% CI, 13.0–61.0%), 59% for scalpel morcellation (95% CI, 33.0–84.0%), and 60% for women in whom no morcellation was used (95% CI, 24.0–98.0%) [17]. In this study, the average age of the group with TH + BSO (n = 90) was higher than that of the group with myomectomy followed by supplemental TH + BSO (n = 72) (47.20 ± 8.94 vs. 40.86 ± 5.88, p < 0.001). It suggests that the choice of surgery may be influenced not only by preoperative evaluation, but also by the age and the desire of fertility. Due to the lack of records, the use of morcellation and specimen retrieval techniques in this study could not be properly analyzed. If the accuracy of preoperative evaluation of lesions can be improved, tumor dispersal can be better avoided. Given that a large proportion of patients were intraoperatively or postoperatively diagnosed of uterine sarcoma, morcellation should be avoided even if preoperative imaging suggests benign uterine fibroids. After having carefully radiologically investigated this type of tumor, en bloc resection of the mass would always be preferable.

The primary limitation of our study is its retrospective nature. A second limitation is that the absence of some records makes some influencing factors incomparable. A third limitation is the limited follow-up period which could not provide information on long-term outcomes. Furthermore, our analysis findings were not validated on an external cohort. We expect to further validate these findings in preoperative assessment and conduct larger scale multicenter prospective studies to verify the accuracy of preoperative diagnosis.

## Conclusions

Analysis of our data showed that a large proportion of uterine sarcomas, especially uLMS and LG-ESS, present mainly with uterine masses. Among the ultrasound features, a large uterine mass diameter and rich hypervascularity may indicate the possibility of uterine sarcoma, especially uLMS. In patients with these ultrasound features, a history of myomectomy may alert clinicians in suspicion of uLMS when compared with LG-ESS.

## Data Availability

The data that support the findings of this study are available from the corresponding author upon reasonable request but restrictions apply to the availability of these data, which were used under license for the current study, and so are not publicly available.

## List of Abbreviations

uLMS: Uterine leiomyosarcoma
LG-ESS: Low-grade endometrial stromal sarcoma
HG-ESS: High-grade endometrial stromal sarcoma
HGUS: High-grade undifferentiated sarcoma
MRI: Magnetic resonance imaging
TH + BSO: Total hysterectomy and bilateral salpingo-oophorectomy
